# The Relative Composition of the Inflammatory Infiltrate as an Additional Tool for Synovial Tissue Classification

**DOI:** 10.1371/journal.pone.0072494

**Published:** 2013-08-08

**Authors:** Cristina Della Beffa, Elisabeth Slansky, Claudia Pommerenke, Frank Klawonn, Jialiang Li, Lie Dai, H. Ralph Schumacher, Frank Pessler

**Affiliations:** 1 Department of Cellular Proteomics, Helmholtz Centre for Infection Research, Braunschweig, Germany; 2 Department of Infection Genetics, Helmholtz Centre for Infection Research, Braunschweig, Germany; 3 Department of Computer Science, Ostfalia University of Applied Sciences, Wolfenbüttel, Germany; 4 Department of Statistics and Applied Probability, National University of Singapore, Singapore, Singapore; 5 Department of Rheumatology, Sun Yat-sen Memorial Hospital, Sun Yat-sen University, Guangzhou, China; 6 Division of Rheumatology, Philadelphia Veteran’s Affairs Medical Center, Philadelphia, Pennsylvania, United States of America; 7 Research Group Biomarkers for Infectious Diseases, TWINCORE Center for Experimental and Clinical Infection Research, Hannover, Germany; INSERM-Université Paris-Sud, France

## Abstract

**Objectives:**

Traditionally, differences in absolute numbers of cells expressing a certain marker (e.g., positive staining cells per mm^2^) have been used in immunohistological synovial tissue classification. We have begun to evaluate the relative composition of the inflammatory infiltrates, i.e. percentages of inflammatory cell types in inflammatory infiltrates, as an alternate classification tool that may potentially improve tissue diagnostics, subgrouping in clinical trials, and understanding of pathogenesis of inflammatory and noninflammatory arthropathies.

**Methods:**

Synovial tissue specimens (normal synovium, n=15; orthopedic arthropathies, n=6; osteoarthritis, n=26; early undifferentiated arthritis, n=10; rheumatoid arthritis, n=26; chronic septic arthritis, n=11) were stained for CD15, CD68, CD3, CD20, and CD38. Densities of cells expressing a given marker were determined in the superficial subintima. Binary and multicategory receiver operating characteristic (ROC) analysis and naïve Bayes classifier were used to compare the abilities of (1) the absolute densities of cells expressing a given marker (absolute method) with (2) the percentages of these cells in the inflammatory cell population (relative method) to differentiate among the six tissue classes.

**Results:**

The inflammatory infiltrates in normal synovium and the orthopedic arthropathies consisted almost exclusively of CD68+ and CD3+ cells. Notable fractions of CD20+ and CD38+ cells appeared in a subset of osteoarthritis samples, and increased further in early, rheumatoid and chronic septic arthritis. ROC analyses and naïve Bayes classifier ranked the absolute method above the relative method in terms of overall discriminatory ability. The relative method became slightly superior when the samples were also stratified according to the total number of inflammatory cells/mm^2^.

**Conclusions:**

This exploratory investigation featuring a variety of joint disorders revealed that measuring the relative proportions of inflammatory cell types may aid in synovial tissue classification if the samples are also stratified according to the intensity of inflammation.

## Introduction

Traditionally, the absolute densities (e.g., cells/mm^2^ of subintimal tissue) of specific inflammatory cell types have been used to classify synovial tissue samples for diagnostic or prognostic purposes, or to measure the response to therapeutic interventions (e.g., [1–8]). While this approach has proved useful in various scenarios, it is conceivable that a look at qualitative features of inflammatory cell populations might provide additional information in specific circumstances. Such an approach might detect the relative contributions of specific arms of the immune system (e.g., innate, cellular or humoral) to synovitis, afford insights into their relative importance to pathogenesis, and provide an additional tool for specific diagnostic scenarios and stratification at the group level in clinical trials. A semi-quantitative scoring method yielding a score from 0 to 4, based on the estimated intensity of infiltration with individual inflammatory cell types, has been published widely and validated by an OMERACT investigator group [9]. This method was also shown to correlate strongly with absolute quantification obtained with digital analysis. However, it does not express the proportion of the entire inflammatory infiltrate that is due to the cell type in question. Moreover, the method is user-dependent and needs to be calibrated separately for each cell type, as their frequencies differ. In other methods the categorical fraction (i.e. 0-5%, 5-25%, etc.) of an inflammatory cell type of all synovial cells is determined, but this approach is limited by the inclusion of fibroblasts and other stromal cells, the proportion of which may be substantial. Using immunohistochemical detection of cell surface markers for the five major inflammatory cell types in synovial infiltrates, we have therefore developed a method based on direct cell counting that yields quantitative data on the fractions of the inflammatory infiltrate due to each of the five cell types. In an exploratory cross-sectional study featuring synovial tissue samples from five types of arthropathies, as well as normal synovium, we have then compared the discriminatory powers of the traditional method based on absolute densities of positive staining cells (absolute method) and the novel approach (referred to as the relative method). We find that the discriminatory accuracy of the traditional, absolute method is superior to that of the relative method for most diagnostic comparisons, but that the relative method leads to some improvement in discriminatory power when the samples are concurrently stratified according to the overall degree of inflammatory infiltration.

## Methods and Materials

The study was approved by the institutional review boards (ethics commitees) of the Philadelphia VA Medical Center, the University of Pennsylvania and Sun Yat-sen Memorial Hospital, Guangzhou, China. All data and specimens were analyzed anonymously, and consent for the current analysis could therefore not be obtained. The pathological synovial tissue samples consisted of closed needle biopsies and surgical specimens from patients with chronic (duration >4 weeks) *septic arthritis* (SeA, n=11), *rheumatoid arthritis* with active disease diagnosed according to the 1987 revised criteria for the classification of rheumatoid arthritis (RA, n=25), *early* undifferentiated *arthritis* (EA, n=10), osteoarthritis (OA, n=26), and “noninflammatory” *orthopedic arthropathies* (Orth.A, n=6). Needle biopsies from healthy volunteers and individuals with non-inflammatory knee pain (n=15) were used as controls (*normal synovium*, denoted N). All specimens have been included in our previous studies of synovial tissue biomarkers and have been characterized extensively with histological and immunohistochemical methods [4–7,10]. These reports also contain representative images of the immunohistological stains for all markers used in the present study. Specifically, the samples used in the present study correspond to a subset of the samples published in ref [7]. for which stains for CD15 (neutrophilic granulocytes), CD68 (macrophages), CD3 (T cells), CD20 (B cells), and CD38 (plasma cells) were available from parallel sections. A semi-automatic immunostainer (Ventana Benchmark, Ventana, Tucson, AZ) with a 24-slide carousel was used. Therefore, more than one staining run was required to complete all stains for a given marker. Demographic and clinical information is summarized per diagnosis in Table 1 and is shown in detail for the RA patients in Table S1. This heterogeneous group comprised patients from Philadelphia receiving disease-modifying anti-rheumatic drugs, and also patients from Guangzhou, China, most of whom were not receiving Western anti-rheumatic treatments despite active disease (Table S1). Due to inherent differences in disease demographics and the limited availability of the EA, SeA and normal specimens, the sample groups differed in terms of demographic variables and the joints of origin. While limiting the ability to generalize from some of the between-group differences, this heterogeneity did allow studying the expression of the five antigens in specimens spanning a broad range of diagnoses and synovitis severity. Moreover, the median synovitis scores of the RA, OA, Orth.A and normal specimens corresponded closely to published reference values for these diagnoses [11], suggesting that these sample groups are representative with regard to the expected degree of synovitis (reference values for EA synovitis are not available, and the score has not been validated for use in SeA).

**Table 1:** Characteristics of patients and tissue samples^a^ .

**Diagnosis**	**N**	**Age (y)**	**Sex, n (%)**	**Synovitis score**	**Joint, n (%)**	**Sample acquisition, n (%)**
SeA	11^b^	56 (7-65)	M, 9 (82)	n/d	Knee, 11 (100)	OR, 11 (100)
			F, 2 (18)			
RA	25^c^	56 (31-80)	M, 12 (50)	5.3 (2.3-8.0)	Knee, 22 (88)	Biopsy, 18 (72)
			F, 12 (50)		Wrist, 3 (12)	Synovect, 7 (28)
EA	10	48 (18-61)	M, 2 (22)	5.1 (2.3-8.0)	Knee, 10 (100)	Biopsy, 10 (100)
			F, 7 (78)			
OA	26	64.5 (41-78)	M, 10 (38)	2.3 (0-4.5)	Knee, 23 (89)	Biopsy, 6 (23)
			F, 16 (62)		Hip, 3 (11)	Arthrosc, 10 (38)
						Arthropl. 10 (38)
Orth. A	6	33 (18-54)	M, 3 (50)	1.5 (0-3)	Knee, 6 (100)	Arthrosc, 6 (100)
			F, 3 (50)			
Normal	15	31 (25-51)	M, 12 (80)	1.2 (0-2.7)	Knee, 15 (100)	Biopsy (10)
			F, 3 (20)			


^a^ Values represent medians (range) unless noted otherwise.

^b^ 11 specimens obtained from 10 patients, since 2 biopsies were available from 1 patient.

^c^ 26 specimens from 25 patients, since 2 biopsies were available from 1 patient.

Abbreviations: Arthropl, arthroplasty; arthrosc, arthroscopy; EA, early arthritis; OA, osteoarthritis; OR, surgical biopsy, synovectomy, or arthroplasty revision; Orth. A, orthopedic arthropathy; RA, rheumatoid arthritis; SeA chronic septic arthritis; synovect, synovectomy.

Inflammatory cell populations were characterized by measuring the densities of cells staining positively for CD15, CD68, CD3, CD20 or CD38 in the synovial subintima to a depth of one 400x microscopic field (0.454 mm) as described previously [4,12]. Cells in extra synovial fibrin deposits were excluded. As in our previous studies, a minimum of 3 needle biopsy pieces were evaluated. In about 50% of the surgical specimens, single tissue pieces were available, which, however, were much larger than the needle biopsies. In all cases, a minimum of 10 high-power microscopic fields were scored per specimen. For each cell type, its absolute density (positive staining cells/mm^2^) or its percentage of the total inflammatory cell (TIC) population (i.e. of the sum of CD15+, CD68+, CD3+, CD20+, and CD38+ cells) was determined and designated “relative cell density”. When comparing more than 2 groups, statistical significance of expression differences was determined in a 2-step procedure, first applying the Kruskall-Wallis analysis and then the Mann–Whitney U test. The Mann–Whitney U test alone was used when comparing differences between two groups. Binary and multicategory receiver operating characteristic (ROC) analysis were carried out using code written in the R environment for statistical computing (http://www.r-project.org/). Multicategory ROC analysis is a recently described extension of ROC analysis that makes it possible to evaluate the discriminatory ability of a test to distinguish among multiple outcomes (diagnoses) [7,13,14]. In this method, the hypervolume under the ROC manifold (HUM) is analogous to the AUC in conventional (binary) ROC analysis but summarizes the accuracy of a test in the simultaneous discrimination among multiple diagnoses. The data mining workbench WEKA [15] was used for naïve Bayes classification. The data set was not normally distributed and exhibited a great variance. Trimmed means were therefore used instead of median values. A trimmed (or truncated) mean is obtained by computing the mean of the samples that remain after a certain percentage of the lowest and highest values (from here on referred to as “trim factor”) has been discarded. For the sample groups with n <12 (Orth.A, EA, and SeA), the mean of the trimmed means with trim factors 10%, 20%, and 30% was computed; and for the sample groups with n ≥12 (N, RA, and OA) the mean of the trimmed means with trim factors 0% (i.e. complete sample), 10%, and 20%.

## Results

### The relative composition of inflammatory infiltrates

**Figure 1:**
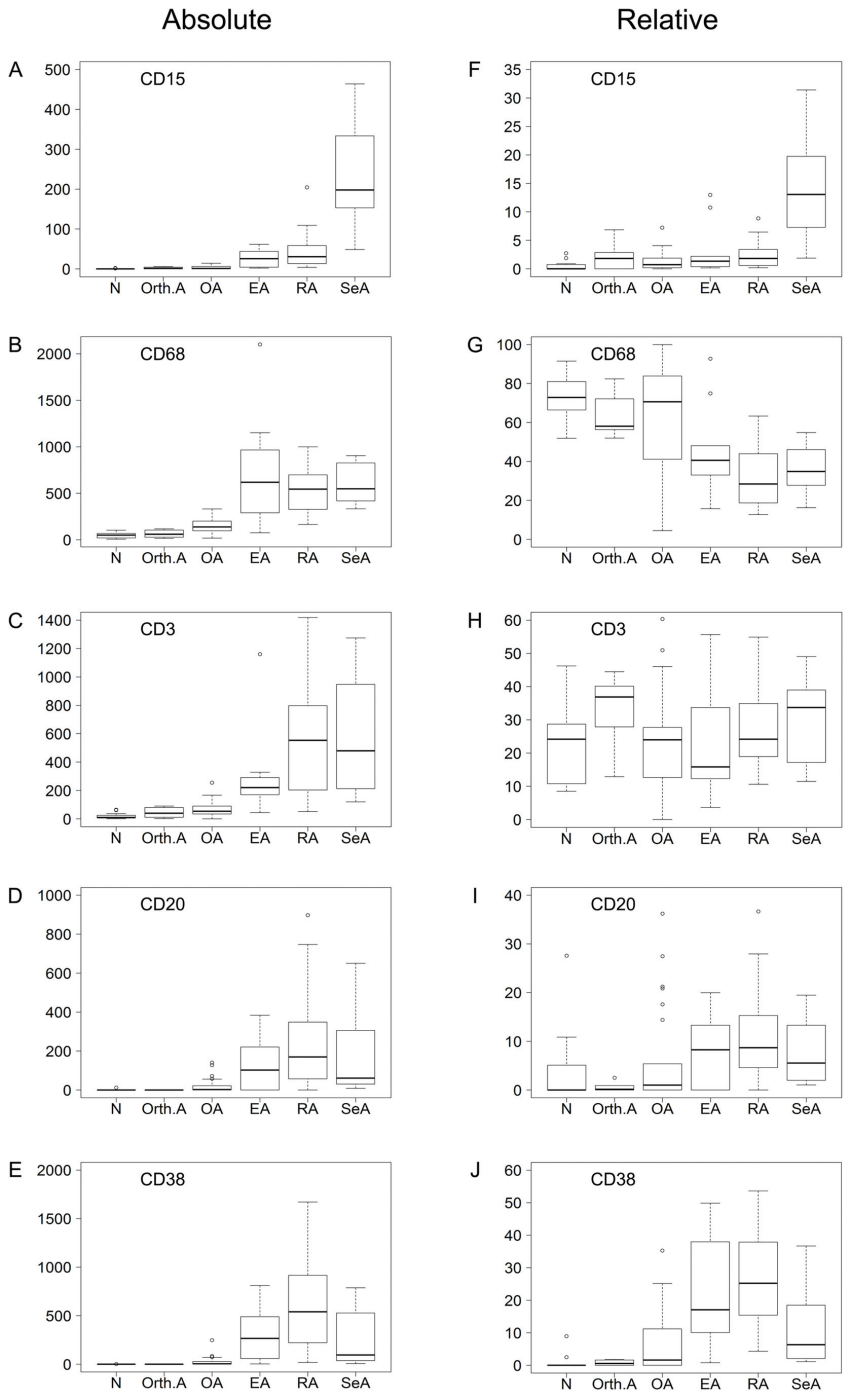
Box plots comparing absolute and relative cell densities in the 6 sample groups. Synovial tissue sections were stained immunohistochemically for CD15, CD68, CD3, CD20, and CD38. The absolute cell densities (left half of the page, labeled “Absolute”, Panels A-E) are expressed as the number of positive staining cells per mm^2^. The relative cell densities (right half of the page, labeled “Relative”, panels F-J) correspond to the cells expressing a given marker as a percentage of the inflammatory cell population, which is defined as the sum of all cells expressing any of the five markers, per mm^2^. Upper and lower borders of the box, 75^th^ percentile and 25^th^ percentiles; horizontal line, median; upper and lower whiskers, maximum and minimum; circles, outliers defined as values above or below the box by a >1.5-fold of the interquartile range.

**Figure 2:**
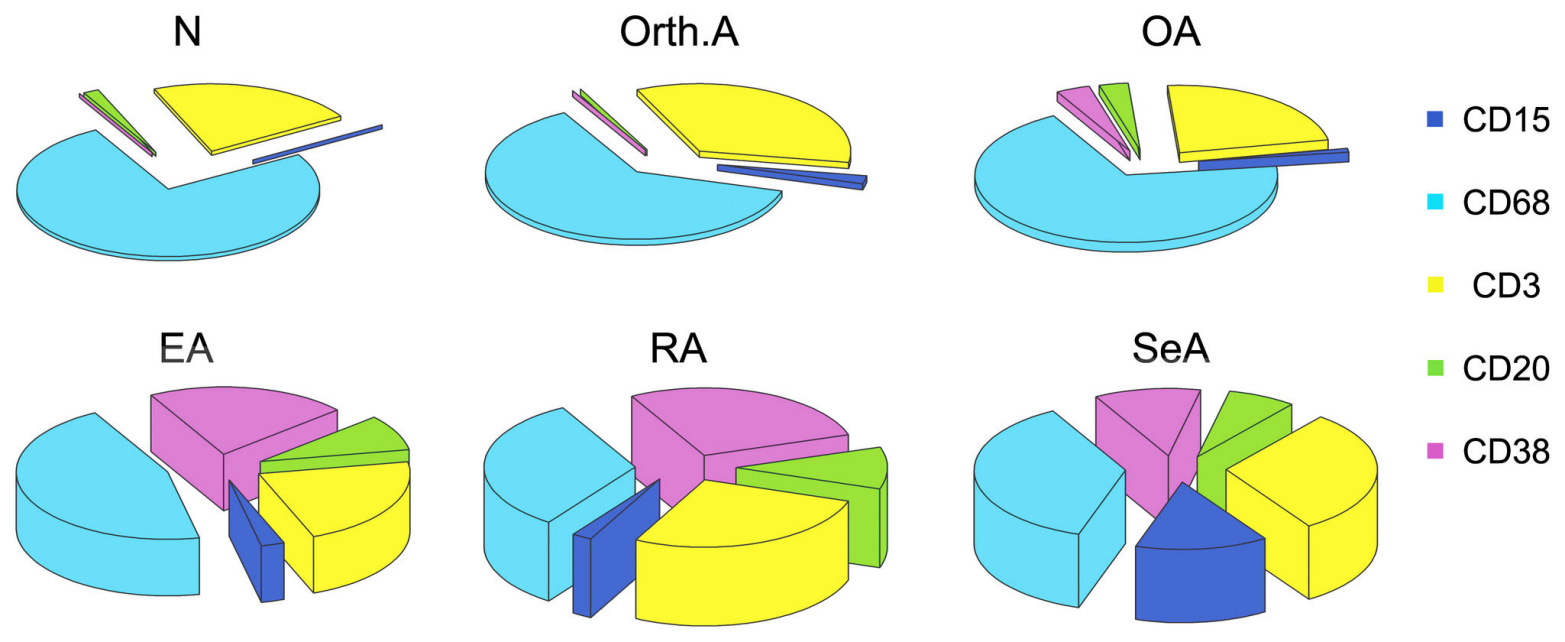
Three-dimensional pie charts illustrating the inflammatory *gestalts* of the arthropathies and normal synovium. The pie sections correspond to the percentages of specific cell types in the inflammatory infiltrates. The height of each pie corresponds to the trimmed mean of the sum of the cells expressing any of the five markers (“total number of inflammatory cells”) and is used to express the overall degree of inflammation.

**Figure 3:**
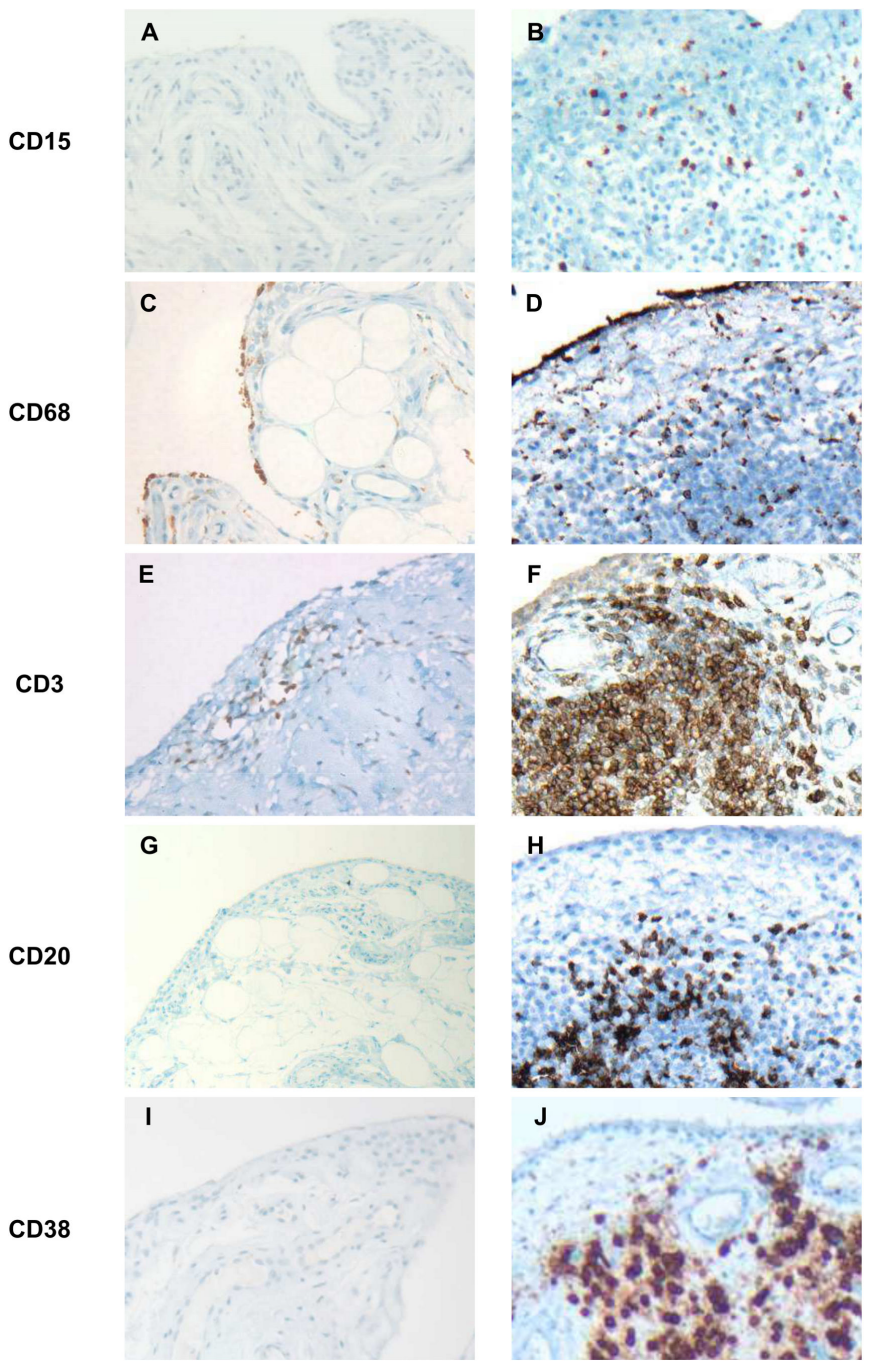
Selected immunohistochemical stains. Less inflamed specimens are arranged in the left column, more inflamed ones in the right column. Stains were done with standard 3-step immunohistochemistry, using diaminobenzidine (DAB) as chromogen (brown) and a hematoxylin counterstain. Original magnifications were 100, 200 or 400x. **A**: Normal synovium demonstrating the complete absence of CD15+ cells typical for this specimen group. **B**: Chronic septic arthritis, exhibiting marked infiltration of CD15+ cells (54CD15+ cells/mm^2^, 2% of total inflammatory cells (TIC). C: Normal synovium with low (25 cells/mm^2^) absolute CD68+ cell density, yet constituting a high proportion (55%) of TIC. **D**: Rheumatoid arthritis (RA) with the characteristic high (573 cells/mm^2^) CD68+ cell density, but representing only 19% of TIC. **E**: Orthopedic arthropathy with low-grade T cell infiltration (88CD3+ cells/mm^2^; 40% of TIC). The remaining 60% of infiltrating cells consisted nearly entirely of CD68+ cells. **F**: RA with high absolute (1409 cells/mm^2^) and relative (55% of TIC) CD3+ cell densities. **G**: Normal synovium demonstrating the characteristic absence of CD20+ cells. **H**: RA specimen with 236CD20+ cells/mm^2^ (9% of TIC). **I**: Normal synovium demonstrating the characteristic absence of CD38+ cells. **J**: RA specimen with particularly CD38-rich infiltrates (absolute density: 1109CD38+ cells/mm^2^; relative density: 41% of TIC).

Figure 1 contrasts the values of the five markers in the six sample groups when either absolute (Panels A-E) or relative (panels F-J) cell densities were used. The absolute cell densities were consistent with those reported in our previous study [7] in that densities of all cell types were markedly higher in the inflammatory arthropathies than in OA, Orth.A or normal tissue. Analysis of the relative cell densities revealed that the inflammatory infiltrates of the normal, Orth.A and OA specimens consisted mainly of cells of cellular immunity, i.e. macrophages and T cells, whereas a considerable contribution from humoral immunity (B cells and plasma cells) appeared only in the chronic inflammatory arthropathies (EA, RA and SeA). CD15+ neutrophils were essentially absent from the normal, Orth.A and OA specimens, but increasing percentages were clearly present in the inflammatory arthropathies, with the highest fraction occurring in SeA. An initial inspection of these data revealed instructive discrepancies between the absolute and relative densities of cells expressing a given marker in one sample group. For instance, the absolute densities of CD68+ and CD3+ cells were low in the normal specimens, but their relative densities were high because few cells expressed any of the other three markers (compare Panels B and C with G and H, respectively). Likewise, the three inflammatory arthropathies contained the highest absolute densities of CD68+ cells, but relative CD68+ cell densities were lower than in the normal and OA specimens due to the higher fractions of CD20+ and CD38+ cells in the 3 chronically inflamed disease states (compare Panels B and G). Combining the relative cell densities with the overall degree of inflammation, as reflected by the total number of inflammatory cells, the inflammatory *gestalts* of the six sample groups could be defined graphically as 3-dimensional pie charts, where the vertical axis reflects the degree of inflammatory infiltration (Figure 2). Selected immunohistochemical stains for the markers are shown in Figure 3.

To quantify differences among the sample groups in infiltration with these cell types more precisely, the six sample groups were then arranged in all possible pairs consisting of one presumably more and one presumably less inflamed sample group. The Kruskall-Wallis test revealed highly significant p values for differences across all diagnoses for all markers except CD3. Statistical significance of the difference between the values in each of the 15 resulting pairs was determined with the Mann Whitney U test (Table S2). Seventy-five p values (i.e. five markers evaluated in 15 paired comparisons) were obtained with the Mann–Whitney U test for the absolute and relative cell densities separately. A much higher number of significant p values (i.e. <0.05) resulted when the absolute cell densities were used (50/75; 66%) than when the relative cell densities were used (30/75; 40%).

**Figure 4:**
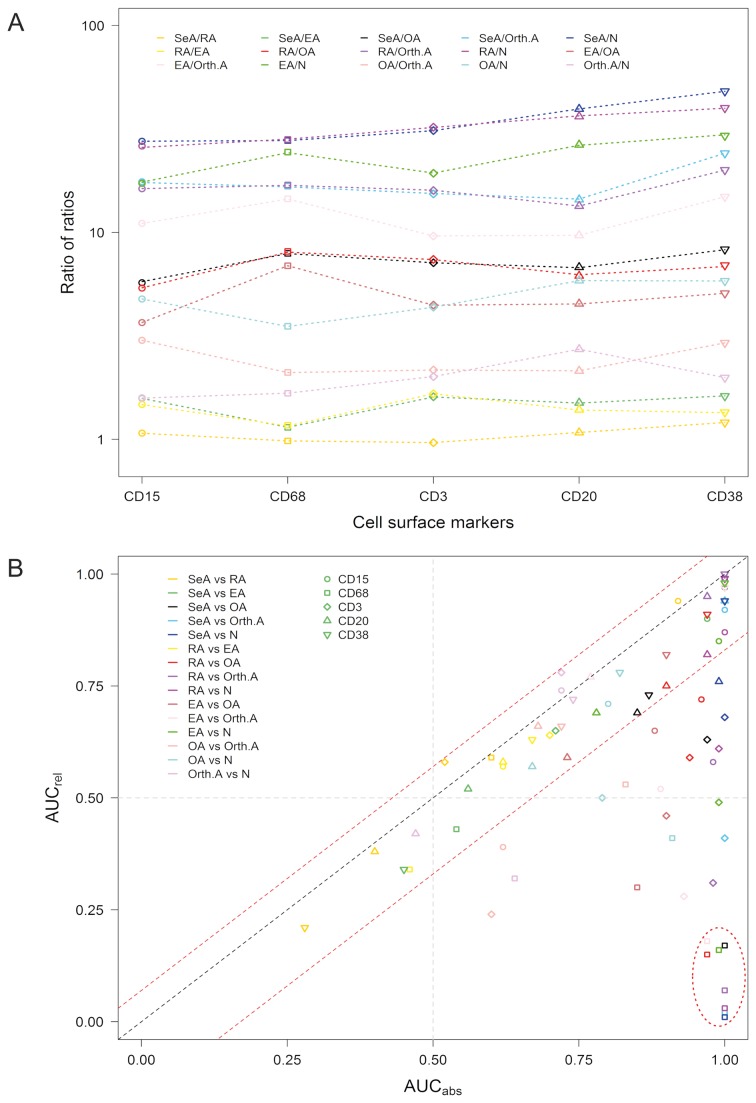
Differences in discriminatory power between the approaches using either the absolute or the relative cell densities. A. Ratio of ratios. Strip chart (1-dimensional scatter plot, log_10_[y]) illustrating discrepancies between the absolute and the relative values in terms of quantitative differences (expressed as ratios) within paired comparisons among the sample groups. Separately for the absolute and the relative values, and for each of the five markers, ratios of expression values were computed for all of the 15 possible paired comparisons among the 6 sample groups, with the presumably more inflamed arthropathy constituting the numerator, resulting in separate expression ratios for the absolute and relative cell densities. Using trimmed means, the ratio of ratios was obtained by the formula: (absolute density_(group 1)_ / absolute density_(group 2)_) / (relative density_(group 1)_ / relative density_(group 2)_). B. Scatter plot illustrating differences in the discriminatory ability of the relative cell densities (y-axis) compared with the discriminatory ability of the absolute cell densities (x-axis). Each data point (symbol) corresponds to the area under the curve (AUC) for the discriminatory power of one marker within one of the 15 possible pairs of sample groups. AUCs were obtained with binary ROC analysis (Table 2). The black line, intercepting the origin, defines all markers where the absolute and relative methods yield identical AUCs. The red lines (with y-intercept equal to 0.07 and -0.17) define threshold bounds, values beyond which define scenarios in which discrepant AUCs resulted when the absolute and the relative cell densities were used (see legend to Supplemental Fig. S1 for details). The symbol shapes identify the markers used, and the symbol colors the sample group pairs to be differentiated, as detailed in the legends inside the graph. The oval line in the lower right quadrant identifies the paired comparisons in which using the relative cell densities resulted in a reversal of the positive state in the ROC analysis (only data points with significant AUCs [*p* <0.05, 95% CI not crossing the midline; Supplemental Table S4] were included).

To facilitate identifying scenarios where absolute and relative cell densities of the same marker differ when their expression is used to differentiate within the same pair of sample groups, the above paired comparisons were set up in such a way that the trimmed mean of the presumably more inflamed sample group constituted the numerator and that of the presumably less inflamed sample group the denominator. The two trimmed means of each pair were then divided by each other, resulting in 150 ratios (75 each for the absolute and relative cell densities). For each of the five markers and the 15 possible paired comparisons, the ratios of trimmed mean expression of the absolute and the relative cell densities were then divided by each other (i.e. ratio (absolute values)/ratio (relative values), yielding 75 “ratio of ratios” values (Figure 4A and Table S3). This analysis showed that the ratios of the relative densities diverged from the ratios of the absolute densities in the majority of the paired comparisons. The highest “ratio of ratios” values (>10; underlined in Table S3) were observed when the highly inflamed sample groups (SeA, RA and EA) were divided by the least inflamed ones (Orth.A or N). The lowest “ratios of ratios” (near 1) resulted with all five markers when SeA was divided by RA, indicating similar differences in absolute and relative expression between these two arthropathies.

### Binary and multicategory ROC analysis

Table 2 summarizes the results of binary ROC analysis based on corrected AUCs (i.e. where AUCs < 0.5 were “corrected” to values >0.5 by transformation to 1-AUC), while Table S4 contains the raw (uncorrected) AUCs. The AUC was used to assess the ability of each of the 5 markers (using either the absolute or relative values) to differentiate between any of the 15 possible pairs of sample groups. In these 75 possible paired comparisons, 50/75 (67%) significant ROC curves (defined as the 95% CI not crossing the midline and p <0.05) resulted when the absolute values were used, and only 29 (39%) when the relative values were used (Table 2). While similar AUCs resulted from some comparisons (e.g. SeA vs RA, SeA vs EA), those derived with the relative cell densities were often lower. When the relative method was used instead of the absolute method, 21 additional AUCs became non-significant (particularly comparisons involving CD3), whereas only one additional AUC became significant (CD38, SeA vs RA). Twenty-three uncorrected AUCs derived with the relative method (8 of which were significant) were below 0.5, reaching values as low as 0.01 (CD68, SeA vs N) (Table S4). In these latter cases, the relative method had led to a reversal of the positive state because the higher expression value was now found in the less inflamed sample group. Overall, there were only two scenarios where the relative method yielded higher AUCs than the absolute method: CD15 and CD38 in the differentiation between SeA and RA (the ROC curves of the relative cell densities were not significant in the few other cases where AUC_rel_ was > AUC_abs_). To test more precisely which of the two methods might be more useful in the overall classification of arthropathies, we then compared the median corrected AUC of the 75 comparisons based on the absolute cell densities with the median corrected AUC of the 75 comparisons made with the relative cell densities. The Kruskall-Wallis rank sum test revealed highly significant differences across all groups (p = 1.9x10^-10^). Indeed, the median corrected AUC of the absolute method was 0.18 AUC higher than that of the relative method (p = 4.9x10^-09^, Mann–Whitney U test; Table 3). Taken together, the above results indicate that the absolute and the relative methods do differ considerably in their discriminatory abilities, both in quantitative and qualitative terms. Interestingly, the sum of all five cell types per mm^2^ (total inflammatory cells, TIC) appeared to have a higher discriminatory power than any of the cell types by themselves (Table 3).

**Table 2:** Results of binary ROC analysis (corrected AUCs).

	
	**CD15**		**CD68**		**CD3**		**CD20**		**CD38**		**TIC**	
	**Abs**	**Rel**	**Abs**	**Rel**	**Abs**	**Rel**	**Abs**	**Rel**	**Abs**	**Rel**		
**SeA : RA**	0.92**	0.94**	0.60	0.59	0.52	0.58	0.60	0.62	0.72	0.79 *	0.52	
**SeA : EA**	0.97**	0.90**	0.54	0.57	0.71	0.65	0.56	0.52	0.55	0.66	0.65	
**SeA : OA**	1.00**	0.97**	1.00**	0.83**	0.97**	0.63	0.85**	0.69	0.87**	0.73	0.99**	
**SeA : Orth.A**	1.00**	0.92*	1.00**	0.98**	1.00**	0.59	1.00**	0.94*	1.00**	0.94*	1.00**	
**SeA : N**	1.00**	0.99**	1.00**	0.99**	1.00**	0.68	0.99**	0.76	1.00**	0.94**	1.00**	
**RA : EA**	0.62	0.57	0.54	0.66	0.70	0.64	0.62	0.58	0.67	0.63	0.67	
**RA : OA**	0.96**	0.72*	0.97**	0.85**	0.94**	0.59	0.90**	0.75*	0.97**	0.91**	0.99**	
**RA : Orth.A**	0.98**	0.58	1.00**	0.93**	0.98**	0.69	0.97**	0.95**	1.00**	1.00**	1.00**	
**RA : N**	1.00**	0.87**	1.00**	0.97**	0.99**	0.61	0.97**	0.82**	1.00**	0.99**	1.00**	
**EA : OA**	0.88**	0.65	0.85**	0.70	0.90**	0.54	0.73	0.59	0.90**	0.82*	0.95**	
**EA : Orth.A**	0.89*	0.52	0.97**	0.82	0.93**	0.72	0.77	0.77	1.00**	0.97*	1.00**	
**EA : N**	0.99**	0.85*	0.99**	0.84*	0.99**	0.51	0.78*	0.69	1.00**	0.98**	1.00**	
**OA : Orth.A**	0.62	0.61	0.83*	0.53	0.60	0.76	0.68	0.66	0.72	0.66	0.83*	
**OA : N**	0.80**	0.71	0.91**	0.59	0.79**	0.50	0.67	0.57	0.82**	0.78**	0.92**	
**Orth.A : N**	0.72	0.74	0.64	0.68	0.72	0.78	0.53	0.58	0.74	0.72	0.67	

Values correspond to AUCs obtained with binary ROC analysis. Corrected AUCs (underlined) were obtained by subtracting all AUCs with an original value of <0.5 from 1. Significant AUCs (marked with asterisks) were defined as (1) CI not crossing the midline (corresponding to an AUC of 0.5), and (2) a p value (adjusted by false discovery rate) of <0.05 (*) or <0.01 (**). Abbreviations: EA, early arthritis; OA, osteoarthritis; Orth.A, orthopedic arthropathy; RA, rheumatoid arthritis; SeA, chronic septic arthritis.

**Table 3:** Quantifying the discriminatory abilities of the absolute and relative cell densities with binary ROC analysis.


		Median AUC^a^			
		Absolute densities		Relative densities	
CD15		0.96 (0.62-1.0)		0.74 (0.52-0.99)
CD68		0.97 (0.54-1.0)		0.82 (0.53-0.99)
CD3		0.93 (0.52-1.0)		0.63 (0.50-0.78)
CD20		0.77 (0.53-1.0)		0.69 (0.52-0.95)
CD38		0.90 (0.55-1.0)		0.82 (0.63-1.0)
All 5 markers^b^		0.90 (0.52-1.0)		0.72 (0.50-1.0)
TIC		0.99 (0.52-1.0)		n/a

^a^ Values correspond to median corrected AUCs (range), which were derived for each marker from the AUCs for the 15 possible paired comparisons shown in Table 2.

^b^ Median of the 75 corrected AUCs that result from applying each of the five markers to the 15 possible paired comparisons. P value for difference in median AUCs across all groups = 1.9x10^-10^ (Kruskall-Wallis test) and for difference between the absolute and relative methods = 4.9x10^-09^ (Mann Whitney U test). Abbreviations: AUC, area under the curve; TIC, total inflammatory cells (sum of CD15+, CD68+, CD38+, CD20+, and CD38+ cells per mm^2^).

Specific discrepancies between the absolute and relative methods were then sought with a scatter plot analysis where, for each marker and each paired comparison, the AUCs that resulted from using the absolute cell densities were plotted against the AUCs using the relative cell densities (Figure 4B). The number of outliers (i.e. scenarios where the absolute and the relative approach yielded divergent AUCs, located below the lower red dotted line in the figure) differed strongly among the five markers: there were none among the 15 paired comparisons when CD38 was used as the discriminatory test, 1/15 (7%) when CD20 was used, 5/15 (33%) when CD15 was used, 11/15 (73%) when CD3 was used and 12/15 (80%) when CD68 was used. Independent of the marker used, results from the absolute and the relative approaches agreed well in the comparisons involving any two of the three inflammatory arthropathies (SeA/RA, SeA/EA, RA/EA). In contrast, in the comparisons involving one inflammatory and one degenerative or the normal sample group (SeA/N, RA/OA, RA/Orth.A, and EA/OA), discrepancies between the two methods were noted in at least 3 of the 5 markers. The 8 comparisons marked with the oval in the lower right quadrant (all involving CD68) correspond to the aforementioned comparisons in which using the relative method led to a reversal of the positive state. Thus, the greatest discrepancies between the absolute and relative approaches resulted when CD68 or CD3 were used as discriminatory markers, or when comparisons were made between inflammatory and non-inflammatory sample groups.

Multicategory ROC analysis was then used to rank the five markers according to the ability to differentiate among all six sample groups simultaneously, using either the absolute or the relative cell densities. The resulting rank list revealed that the HUMs (i.e. the measure of discriminatory power that is used in multicategory ROC analysis) were usually greater when absolute cell densities were used than when relative values were used (Table 4, Column 3). Consistent with the above observation that TIC had a greater discriminatory power than any of the markers alone (Table 3), the HUM of TIC was higher than that of any of the absolute or relative densities of the 5 cell types. When combined with TIC, the relative cell densities of 3 of the 5 markers reached a slightly higher overall discriminatory ability than the respective absolute densities (Table 4, Column 5).

**Table 4:** Multicategory ROC analysis: ranking the absolute and relative cell densities according to overall discriminatory ability.

**Rank**	**Marker alone**	**HUM^1^**	**Marker combined with TIC^2^**	**HUM**
1	TIC	0.118	TIC/CD15_rel_^3^	0.142
2	CD68	0.106	TIC/CD15	0.140
3	CD15	0.087	TIC/CD68_rel_	0.128
4	CD38	0.082	TIC/CD3	0.123
5	CD3	0.070	TIC/CD38 _rel_	0.122
6	CD68 _rel_	0.044	TIC/CD38	0.121
7	CD38 _rel_	0.037	TIC/CD20 _rel_	0.119
8	CD15 _rel_	0.032	TIC/CD3 _rel_	0.119
9	CD20	0.018	TIC/CD20	0.119
10	CD3 _rel_	0.0091	TIC/CD68	0.118
11	CD20 _rel_	0.0090	TIC	0.118
12	Null hypothesis^4^	0.0014	Null hypothesis^4^	0.0014

^1^ HUM, hypervolume under the manifold, calculated according to ref. [12].

^2^ TIC, total inflammatory cells (sum of CD3+, CD20+, CD38+, CD68+, and CD15+ cells per mm^2^).

^3^ Subscript rel: relative cell density of the respective marker. Absolute cell densities in all other cases.

^4^ HUM of a hypothetical nondiscriminatory marker, considering the number of classes and the presence of ties in this analysis.

### Naïve Bayes classifier

**Figure 5:**
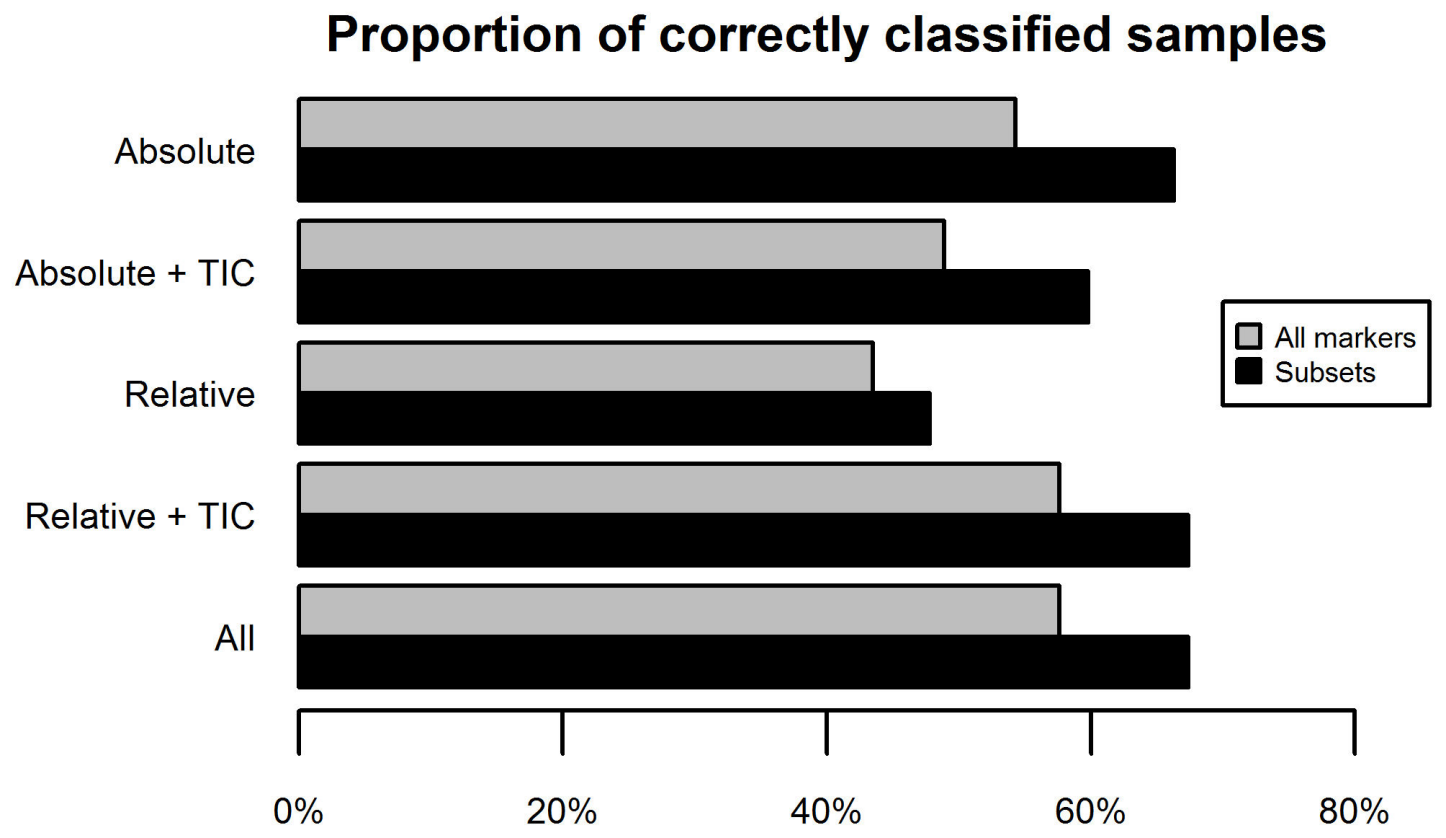
Proportion of correctly classified disease states for the various marker categories as determined with the Bayes classifier. **Grey bars**: prediction with complete sets. The “Relative” set comprised the relative values of CD15+, CD68+, CD3+, CD20+, or CD38+ cells. The set “Relative + TIC” comprises the “Relative” set plus total inflammatory cells (TIC, sum of absolute expression values of all five markers). The “Absolute” set comprised absolute densities (cells/mm^2^) of cells expressing CD15+, CD68+, CD3+, CD20+, or CD38+ cells. The set “Absolute + TIC” comprised the “Absolute” set plus TIC. **Black bars**: wrapper-predicted subsets of markers. “Relative”: relative densities of CD15+, CD38+, or CD68+ cells. “Relative + TIC” relative densities of CD15+ or CD68+ cells, plus TIC. Both the “Absolute” and “Absolute + TIC” sets comprised absolute values of CD15+, CD38+, or CD68+ cells. Abbreviation: TIC, total inflammatory cells.

In order to assess the robustness of the markers to classify the tissue sample groups correctly, we applied the naïve Bayes classifier to the data set (Figure 5). The percentage of correctly predicted patients was determined based on the leave-one-out (jackknife) cross-validation method. As shown by the grey bars in the figure, the absolute values of the markers yielded the highest percentage of correctly identified patients (58%), followed by the combination of TIC with the relative values (49%) and, last, the relative values alone (44%). Adding TIC to the absolute values did not improve disease diagnosis prediction, thus supporting the results of the multicategory ROC analysis. Aiming to extract the key markers (attributes) responsible for correct classification we then applied a wrapper method in combination with the naïve Bayes classifier. For the attribute category 'Relative' this approach identified the relative densities of CD15+ CD38+, and CD68+ cells as the key markers. When TIC was included along with the relative values, it was identified as a key marker instead of CD38. In the 'Absolute' category, this analysis selected CD15, CD68, and CD38 as key markers, which persisted even when TIC was included. Applying the Bayes classifier on these subsets only increased the accuracy of all attribute categories (black bars). Confusion matrices of the combination of different markers are shown in Table 5. The highest accuracy (62 patients = 67%; Panel A) was achieved with the combination CD15/CD68/CD38. Specifically, the subset consisting of CD15_abs_, CD68_abs_ and CD38_abs_ (i.e. the absolute densities of cells expressing these markers) correctly classified 14 OA and four EA patients more than the subset consisting of CD15_rel_, CD38_rel_ and CD68_rel_ (Panel B). Interestingly, when TIC was substituted for CD38_rel_, these differences diminished to two OA and three EA patients, whereas two additional RA patients were classified correctly (Panel C). However, this subset still achieved 7 fewer correctly predicted disease classifications than the triad CD15_abs_, CD68_abs_, and CD38_abs_. Thus, as seen in the multicategory ROC analysis (Table 3), adding TIC improved overall classification accuracy of the relative cell markers.

**Table 5:** Confusion matrices (naïve Bayes classifier).


A. Absolute method								B. Relative method							C. Relative method + TIC					
	N	Orth.A	OA	EA	RA	SeA		N	Orth.A	OA	EA	RA	SeA		N	Orth.A	OA	EA	RA	SeA
**N**	14	1	0	0	0	0		13	1	1	0	0	0		11	2	2	0	0	0
**Orth.A**	4	0	2	0	0	0		3	1	1	1	0	0		2	0	3	1	0	0
**OA**	5	1	19	1	0	0		13	3	5	0	5	0		6	1	17	0	2	0
**EA**	0	0	0	4	6	0		1	0	2	0	5	2		0	0	0	1	7	2
**RA**	0	0	3	3	17	1		0	0	5	0	18	1		0	0	4	1	19	0
**SeA**	0	0	0	0	3	8		0	1	0	0	3	7		0	0	0	1	3	7

The sum of the values in the diagonal of the matrix gives the number of correctly classified patients: A, 62/92 (67.4%). B, 44/92 (47.8%). C, 54/92 (59.8%). Abbreviations: EA, early arthritis; OA, osteoarthritis; Orth.A, orthopedic arthropathy; RA, rheumatoid arthritis; SeA, chronic septic arthritis.

## Discussion

We have evaluated the usefulness of the relative proportions of five major inflammatory cell types in the inflammatory infiltrates of synovitis in synovial tissue classification.

### Composition of the inflammatory infiltrates

Firstly, comparing these cell fractions among the six sample groups, distinct qualitative differences were detected: the near absence of neutrophilic granulocytes and humoral immune cells in normal synovium, the predominance of cellular immunity in the infiltrates of the orthopedic arthropathies, the emergence of humoral immune cells in a subset of OA synovia, the general presence of large fractions of humoral immune cells in RA, and the gradual increase of neutrophilic granulocyte fractions from OA over RA to SeA. These results are consistent with previous results by us [5,12] and others [16], but represent –to our knowledge- the first such analysis among several synovial sample groups, defined by distinct clinical diagnoses, in the same study. The pie charts in Figure 2 were used to express the inflammatory *gestalts* of the sample groups. This approach might serve as a simple visual tool to express qualitative and quantitative aspects of a defined group of synovial tissue specimens. Indeed, we are using it in our ongoing studies of less common arthropathies that have previously not been characterized immunohistologically (Ogdie and Pessler, unpublished data). We used the sum of all immunohistochemically detected infiltrating cells (TIC) to define the overall degree of inflammation, i.e. the vertical axis. Conceivably, a standard histologic measure of synovitis, such as the 3-component synovitis score according to Krenn et al. [14,17], which measures lining hyperplasia and stromal density in addition to inflammatory infiltration, could be used to define this axis.

### Usefulness of the relative proportions of inflammatory cells in synovial tissue classification

Both binary (Tables 2 and 3) and multicategory (Table 4) ROC analyses clearly showed that, overall, using the absolute cell densities was superior to using the relative cell densities for the purpose of synovial tissue classification. Indeed, there were only two diagnostic scenarios in which the relative method was somewhat better than the absolute method. However, the relative method became more powerful when the tissues were also stratified according to the overall degree of inflammation. This notion is also supported by the results obtained with the naïve Bayes classifier (Figure 5). Taken together, these results suggest that any application of the relative composition of the inflammatory infiltrates for synovial tissue classification should also include a general measure of inflammation. As mentioned in the previous paragraph, a synovitis score could be used for this purpose instead of TIC. Alternatively, specific biomarkers of inflammation in synovial membrane or fluid, such as cytokines or chemokines, might prove useful for this purpose.

### Potential applications

Immunohistological markers have been used to classify synovial tissue specimens for diagnostic purposes [1,7], but also to identify pretreatment tissue markers that predict a treatment response [3], and to identify markers that correlate best with successful treatment [2]. Measuring the relative cell densities would consider other, potentially important, aspects of changes in the composition of inflammatory cell populations, such as selective changes in specific cell populations while controlling for the overall degree of inflammation. Such data might, for instance, become useful in selected difficult diagnostic scenarios or in prospective studies where (in contrast to the presented study) both pre- and post-treatment biopsies and/or correlations which changes in clinical or radiographic disease activity are available.

### Use of multicategory ROC analysis

The present study also underscores the usefulness of multicategory ROC analysis in ranking several markers (tests) in the ability to differentiate among several outcomes (diagnoses). After its initial description as a biostatistical tool for tumor classification with oligonucleotide microarray data [13], we have used multicategory ROC analysis to rank immunohistochemical markers of synovitis according to their discriminatory power [7], and to measure the relative contribution of each of the three components of the Krenn synovitis score [17] to its diagnostic accuracy [14]. In the presented study, it enabled us to establish rank lists of the 5 markers either without or combined with TIC, and this approach revealed the gain in overall discriminatory ability when the relative values were combined with TIC.

### Limitations

This study is limited by the fact that the densities of positive staining cells were determined with manual cell counting. This method is accurate and comparable to digital image analysis [18], but it is too cumbersome for routine use in diagnostic practice or studies with larger sample sizes. However, since quantification of positive staining cells with digital image analysis is well established, our approach should also be feasible with digitally analyzed tissue sections. The analysis was limited to the major inflammatory cell types for which immunostains are available at most institutions. Less common cell types, notably dendritic and natural killer cells, were not included, leaving the possibility that their inclusion might have changed results to some extent. In addition, sample sizes of the Orth.A, EA and SeA groups were relatively small, and the selection of clinical diagnoses did not include other important arthropathies such as spondylarthritis or reactive arthritis, or comparisons of prospectively collected consecutive biopsies from defined patient cohorts. Moreover, due to the heterogeneity in diagnoses, the synovial tissue samples had to be obtained during a variety of interventions (synovectomy, needle biopsy, arthroplasty, arthroscopy) and from joints at various anatomical sites. Therefore, tissue could not be sampled from the same site in all cases, likely creating some bias. Thus, validation of the results in studies featuring homogeneous patient populations and standardized tissue sampling procedures will be important.

## Supporting Information

Figure S1 **Differences between AUCs obtained using the absolute (AUC_abs_) or relative (AUC_rel_) cell densities.**
For each of the 75 comparisons (five markers and 15 pairs of sample groups; plotted on the x-axis) the difference between AUC_abs_ and AUC_rel_ was computed and plotted on the y-axis. The black dotted line corresponds to the hypothetical condition where AUC_abs_ = AUC_rel_. Fifty-two values (69%) fell into the interval between -0.07 and 0.17 (red interrupted lines). Values above 0.17 were considered outliers, identifying scenarios in which discrepant results were obtained with the absolute vs. the relative method. These corresponded to (AUC_abs_ - AUC_rel_) values between -0.06 and 0.99. Two threshold lines were then drawn in Figure 4B, according to the formula AUC_abs_ – AUC_rel_ = constant; in mathematical terms x – y = c → y = x – c, with c = 0.17 and c = -0.07.
(TIF)

Table S1 **Demographic and clinical data of the RA patients (n=25)**.
(DOCX)

Table S2 P values for differences in trimmed mean expression values.
(DOCX)

Table S3 Ratio of ratios of trimmed means of cell densities.
(DOCX)

Table S4 **Results of binary ROC analysis (uncorrected AUCs)**.
(DOCX)
