# Supplementary figures and images for: The Relative Composition of the Inflammatory Infiltrate as an Additional Tool for Synovial Tissue Classification
Source: PLoS One. 2013 Aug 8;8(8):e72494. doi: 10.1371/journal.pone.0072494 (PMC3738641; doi:10.1371/journal.pone.0072494)

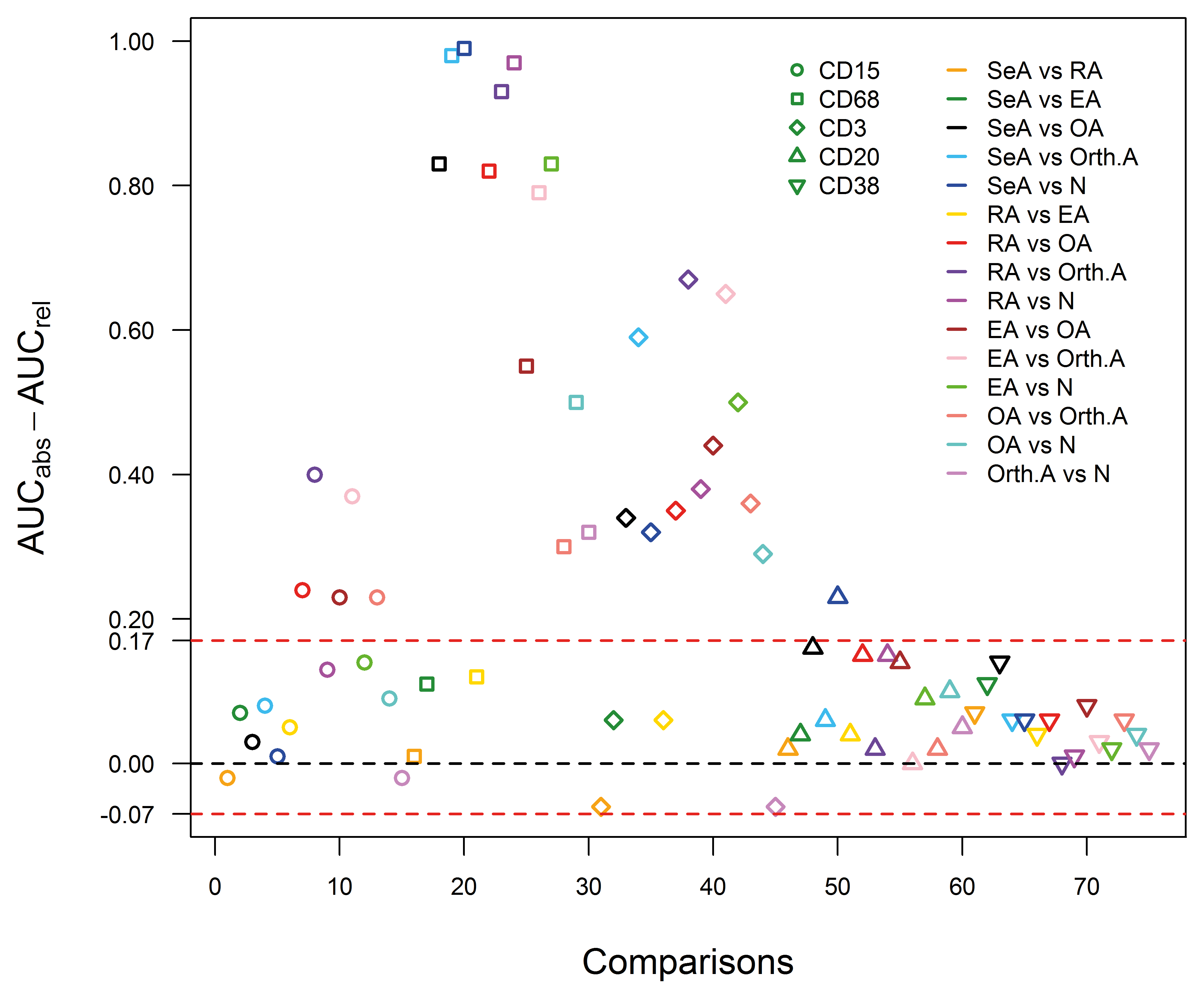

Supplement: Figure S1 — Differences between AUCs obtained using the absolute (AUCabs) or relative (AUCrel) cell densities. For each of the 75 comparisons (five markers and 15 pairs of sample groups; plotted on the x-axis) the difference between AUCabs and AUCrel was computed and plotted on the y-axis. The black dotted line corresponds to the hypothetical condition where AUCabs = AUCrel. Fifty-two values (69%) fell into the interval between -0.07 and 0.17 (red interrupted lines). Values above 0.17 were considered outliers, identifying scenarios in which discrepant results were obtained with the absolute vs. the relative method. These corresponded to (AUCabs - AUCrel) values between -0.06 and 0.99. Two threshold lines were then drawn in Figure 4B, according to the formula AUCabs – AUCrel = constant; in mathematical terms x – y = c → y = x – c, with c = 0.17 and c = -0.07. (TIF) [file pone.0072494.s001.tif]
