# Supplementary material for: The Relative Composition of the Inflammatory Infiltrate as an Additional Tool for Synovial Tissue Classification
Source: PLoS One. 2013 Aug 8;8(8):e72494. doi: 10.1371/journal.pone.0072494 (PMC3738641; doi:10.1371/journal.pone.0072494)
Supplement: Table S1 — Demographic and clinical data of the RA patients (n=25). (DOCX) [file pone.0072494.s002.docx]

| **Supplemental Table S1.** Demographic and clinical data of the RA patients (n=25) | | | | | | | | | | | | | | |  |  |
| --- | --- | --- | --- | --- | --- | --- | --- | --- | --- | --- | --- | --- | --- | --- | --- | --- |
| **Pt** | **Age** | **Ethnicity** | **Sex** | **Dura-tion (mo)** | **RF** | **ESR^1^** | **Medi-**  **cations^2^** | **Sample**  **acqui-sition^3^** | **Joint^4^** | | **DAS28** | | **Syno-**  **vitis**  **score^5^** | |  |  |
| 1 | 49 | AA | F | 0,3 | + | 80 | M, H | BX | LK | | 6,8 | | 5,5 | |  |  |
| 2 | 80 | AA | M | 36 | + | n/d | NSAID | BX | RK | | n/d | | 6,9 | |  |  |
| 3 | 66 | AA | M | n/d | + | n/d | NSAID | BX | LW | | n/d | | 5,0 | |  |  |
| 4 | 68 | Cauc | M | 24 | + | 60 | M, P | BX | LK | | 3,0 | | 2,3 | |  |  |
| 5 | 68 | Cauc | M | n/d | + | n/d | M, P, Vald | BX | LK | | n/d | | 5,0 | |  |  |
| 6 | 66 | Cauc | M | 132 | + | 124 | M, Gold, P | BX | RK | | 5,5 | | 5,0 | |  |  |
| 7 | 53 | Cauc | M | 12 | + | 43 | H | BX | LK | | 5,0 | | 7,1 | |  |  |
| 8 | 53 | Cauc | M | 13 | + | 50 | H, V | BX | LK | | 5,0 | | 6,5 | |  |  |
| 9 | 31 | Cauc | M | 132 | n/d | n/d | E, L, P | SYN | LK | | n/d | | 6,5 | |  |  |
| 10 | 68 | Cauc | M | 312 | + | 56 | E, L, H, P | SYN | RW | | 6,3 | | 5,3 | |  |  |
| 11 | 80 | AA | M | 120 | + | 67 | E, M, P | SYN | RW | | 7,6 | | 6,1 | |  |  |
| 12 | 44 | Han | M | 120 | + | 82 | M, P, L | SYN | RK | | n/d | | 6,5 | |  |  |
| 13 | 48 | Han | F | 36 | + | 107 | NSAID | SYN | RK | | 5,5 | | 7,0 | |  |  |
| 14 | 39 | Han | F | 36 | + | n/d | TOM^6^ | SYN | LK | | 2,6 | | 7,3 | |  |  |
| 15 | 48 | Han | F | 360 | + | 41 | P | SYN | RK | | 7,6 | | 4,5 | |  |  |
| 16 | 43 | Han | F | 60 | + | 74 | IA | BX | RK | | 7,6 | | 4,0 | |  |  |
| 17 | 74 | Han | F | 360 | - | 62 | TOM | BX | LK | | 7,7 | | 3,0 | |  |  |
| 18 | 54 | Han | F | 168 | + | 85 | M, TOM | BX | LK | | 6,1 | | 5,5 | |  |  |
| 19 | 53 | Han | F | 120 | + | 1 | TOM | BX | LK | | 5,7 | | 2,5 | |  |  |
| 20 | 62 | Han | F | 18 | + | 88 | P | BX | RK | | 7,1 | | 4,0 | |  |  |
| 21 | 34 | Han | F | 120 | - | 27 | TOM | BX | LK | | 5,0 | | 6,5 | |  |  |
| 22 | 42 | Han | F | 8 | + | 35 | NSAID, TOM | BX | LK | | 4,1 | | 8,0 | |  |  |
| 23 | 68 | Han | F | 120 | + | 104 | P | BX | RK | | 5,4 | | 3,5 | |  |  |
| 24 | 34 | Han | F | 48 | + | 67 | n/d | BX | FK | | 4,5 | | 3,0 | |  |  |
| 25 | 70 | Han | M | 72 | + | 88 | NSAID | BX | RK | | 6,5 | | 5,0 | |  |  |
| ^1^Value within 2 weeks of procedure. | | | | | |  | | | | | | | | |  |  |
| ^2^Chronic rheumatologic medications taken at the time of biopsy or synovectomy. Abbreviations: E, etanercept;  H, hydroxychloroquine; IA, intraarticular corticosteroid injections; L, leflunomide; M, methotrexate; n/d, not determined; P, prednisone; TOM, traditional Oriental medicine; Vald., valdecoxib. | | | | | | | | | | | | | | | | |
| ^3^Technique used to obtain tissue. BX, closed needle biopsy; SYN, synovectomy.  ^4^Joint from which the synovial tissue was obtained: L, left; K, knee; R, right; W, wrist. | | | | | | | | | |  | |  | |  |  |  |
| ^5^Synovitis score according to Krenn et al. [14,17]. Three features of synovitis (intimal hyperplasia, stromal  density, inflammatory infiltration) are each assigned a score from 0 to 3, yielding a maximum score of 9.  ^6^Traditional Oriental medicine. | | | | | | | | | | | | | | | | |
