# Supplementary material for: The Relative Composition of the Inflammatory Infiltrate as an Additional Tool for Synovial Tissue Classification
Source: PLoS One. 2013 Aug 8;8(8):e72494. doi: 10.1371/journal.pone.0072494 (PMC3738641; doi:10.1371/journal.pone.0072494)
Supplement: Table S2 — P values for differences in trimmed mean expression values. (DOCX) [file pone.0072494.s003.docx]

| **Supplemental Table S2.** P values for differences in trimmed mean expression values | | | | | | | | | | |
| --- | --- | --- | --- | --- | --- | --- | --- | --- | --- | --- |
| **Comparison** | **CD15** | | **CD68** | | **CD3** | | **CD20** | | **CD38** | |
|  | **Abs** | **Rel** | **Abs** | **Rel** | **Abs** | **Rel** | **Abs** | **Rel** | **Abs** | **Rel** |
| **SeA : RA** | 2.3x10^-4^ | 3.9x10^-4^ | 0.42 | 0.49 | 0.90 | 0.53 | 0.38 | 0.36 | 0.05 | 0.02 |
| **SeA : EA** | 7.4x10^-4^ | 8.6x10^-3^ | 0.83 | 0.65 | 0.14 | 0.34 | 0.69 | 0.95 | 0.74 | 0.33 |
| **SeA : OA** | 1.4x10^-5^ | 1.7x10^-4^ | 1.4x10^-5^ | 8.6x10^-3^ | 4.6x10^-5^ | 0.33 | 1.6x10^-3^ | 0.13 | 8.9x10^-4^ | 0.07 |
| **SeA : OrthA** | 2.0x10^-3^ | 0.02 | 2.0x10^-3^ | 7.1x10^-3^ | 2.0x10^-3^ | 0.65 | 2.0x10^-3^ | 0.01 | 2.0x10^-3^ | 0.01 |
| **SeA : N** | 5.5x10^-5^ | 2.1x10^-4^ | 8.2x10^-5^ | 3.1x10^-4^ | 8.2x10^-5^ | 0.21 | 9.0x10^-5^ | 0.07 | 3.1x10^-5^ | 4.9x10^-4^ |
| **RA : EA** | 0.35 | 0.63 | 0.75 | 0.27 | 0.10 | 0.33 | 0.32 | 0.58 | 0.15 | 0.33 |
| **RA : OA** | 6.0x10^-7^ | 0.02 | 6.0x10^-7^ | 2.1x10^-4^ | 1.9x10^-6^ | 0.37 | 7.6x10^-6^ | 0.01 | 6.0x10^-7^ | 2.5x10^-5^ |
| **RA : OrthA** | 8.9x10^-4^ | 0.65 | 6.1x10^-4^ | 7.1x10^-3^ | 9.6x10^-4^ | 0.27 | 1.1x10^-3^ | 4.3x10^-3^ | 6.1x10^-4^ | 1.3x10^-3^ |
| **RA : N** | 2.2x10^-6^ | 8.8x10^-4^ | 2.4x10^-6^ | 2.5x10^-5^ | 2.8x10^-6^ | 0.36 | 7.6x10^-6^ | 4.3x10^-3^ | 1.9x10^-6^ | 1.6x10^-5^ |
| **EA : OA** | 1.3x10^-3^ | 0.30 | 2.5x10^-3^ | 0.13 | 7.4x10^-4^ | 0.79 | 0.05 | 0.49 | 6.1x10^-4^ | 0.01 |
| **EA : OrthA** | 0.02 | 0.98 | 4.7x10^-3^ | 0.10 | 9.1x10^-3^ | 0.29 | 0.10 | 0.14 | 2.4x10^-3^ | 0.01 |
| **EA : N** | 9.0x10^-5^ | 0.01 | 1.9x10^-4^ | 0.01 | 1.9x10^-4^ | 0.99 | 0.02 | 0.18 | 4.5x10^-5^ | 2.1x10^-4^ |
| **OA : OrthA** | 0.43 | 0.51 | 0.02 | 0.91 | 0.53 | 0.12 | 0.21 | 0.33 | 0.13 | 0.33 |
| **OA : N** | 2.0x10^-3^ | 0.05 | 7.4x10^-5^ | 0.47 | 3.4x10^3^ | 0.99 | 0.10 | 0.51 | 8.2x10^-4^ | 7.1x10^-3^ |
| **OrthA : N** | 0.12 | 0.13 | 0.38 | 0.33 | 0.16 | 0.12 | 0.88 | 0.65 | 0.05 | 0.12 |
| Values correspond to p values (Mann Whitney U test) for differences in trimmed mean expression of any of the 5 markers in any of the 15 possible pairs of diagnoses. Pairs are set up such that the presumably more inflamed sample group (diagnosis) is listed first, using the hierarchy SeA>RA>EA>OA>Orth.A>N. P values (adjusted for FDR) obtained with Kruskall-Wallis analysis across all comparisons featuring each one the 5 surface markers: CD15_abs_, 1.0x10^-12^; CD15_rel_, 3.3x10^-7^; CD68_abs_, 1.8x10^-12^; CD68_rel_, 1.6x10^-7^; CD3_abs_, 8.4x10^-12^; CD3_rel_, 0.22; CD20_abs_, 1.4x10^-8^; CD20_rel_, 8.3x10^-4^; CD38_abs_, 2.1x10^-12^; CD38_rel_, 3.5x10^-10^.  Abbreviations: abs, absolute cell densities; EA, early arthritis; FDR, false discovery rate; OA, osteoarthritis; Orth.A, orthopedic arthropathy; RA, rheumatoid arthritis; rel, relative cell densities; SeA, septic arthritis. | | | | | | | | | | |
