# Supplementary material for: The Relative Composition of the Inflammatory Infiltrate as an Additional Tool for Synovial Tissue Classification
Source: PLoS One. 2013 Aug 8;8(8):e72494. doi: 10.1371/journal.pone.0072494 (PMC3738641; doi:10.1371/journal.pone.0072494)
Supplement: Table S3 — Ratio of ratios of trimmed means of cell densities. (DOCX) [file pone.0072494.s004.docx]

| **Supplemental Table S3.** Ratio of ratios of trimmed means of cell densities. | | | | | |
| --- | --- | --- | --- | --- | --- |
| **Comparison** | **CD15** | **CD68** | **CD3** | **CD20** | **CD38** |
|  | **Abs/Rel** | **Abs/Rel** | **Abs/Rel** | **Abs/Rel** | **Abs/Rel** |
| **SeA/RA** | 1.07 | 0.98 | 0.96 | 1.08 | 1.21 |
| **SeA/EA** | 1.58 | 1.14 | 1.60 | 1.50 | 1.62 |
| **SeA/OA** | 5.78 | 7.90 | 7.13 | 6.76 | 8.25 |
| **SeA/Orth.A** | 17.4 | 16.6 | 15.4 | 14.5 | 24.2 |
| **SeA/N** | 27.6 | 27.8 | 31.1 | 39.5 | 48.1 |
| **RA/EA** | 1.47 | 1.16 | 1.66 | 1.39 | 1.35 |
| **RA/OA** | 5.40 | 8.04 | 7.39 | 6.25 | 6.84 |
| **RA/Orth.A** | 16.3 | 16.9 | 16.0 | 13.4 | 20.0 |
| **RA/N** | 25.8 | 28.3 | 32.2 | 36.6 | 39.9 |
| **EA/OA** | 3.67 | 6.91 | 4.45 | 4.51 | 5.08 |
| **EA/Orth.A** | 11.1 | 14.5 | 9.6 | 9.7 | 14.9 |
| **EA/N** | 17.5 | 24.3 | 19.4 | 26.4 | 29.6 |
| **OA/Orth.A** | 3.01 | 2.10 | 2.16 | 2.15 | 2.93 |
| **OA/N** | 4.77 | 3.52 | 4.35 | 5.85 | 5.83 |
| **Orth.A/N** | 1.58 | 1.67 | 2.01 | 2.73 | 1.99 |
| Pairs are set up such that the presumably more inflamed sample group (diagnosis) constitutes the  numerator, using the hierarchy SeA>RA>EA>OA>Orth.A>N. Ratios >10 are underlined.  Abbreviations: EA, early arthritis; OA, osteoarthritis; Orth.A, orthopedic arthropathy; RA, rheumatoid  arthritis; SeA, chronic septic arthritis. | | | | | |
