# Supplementary material for: The Relative Composition of the Inflammatory Infiltrate as an Additional Tool for Synovial Tissue Classification
Source: PLoS One. 2013 Aug 8;8(8):e72494. doi: 10.1371/journal.pone.0072494 (PMC3738641; doi:10.1371/journal.pone.0072494)
Supplement: Table S4 — Results of binary ROC analysis (uncorrected AUCs). (DOCX) [file pone.0072494.s005.docx]

| **Supplemental Table S4.** Results of binary ROC analysis (uncorrected AUCs). | | | | | | | | | | | |
| --- | --- | --- | --- | --- | --- | --- | --- | --- | --- | --- | --- |
|  | **CD15** | | **CD68** | | **CD3** | | **CD20** | | **CD38** | | **TIC** |
|  | **Abs** | **Rel** | **Abs** | **Rel** | **Abs** | **Rel** | **Abs** | **Rel** | **Abs** | **Rel** |  |
| **SeA : RA** | 0.92** | 0.94** | 0.60 | 0.59 | 0.52 | 0.58 | 0.40 | 0.38 | 0.28 | 0.21* | 0.48 |
| **SeA : EA** | 0.97** | 0.90** | 0.54 | 0.43 | 0.71 | 0.65 | 0.56 | 0.52 | 0.45 | 0.34 | 0.65 |
| **SeA : OA** | 1.00** | 0.97** | 1.00** | 0.17** | 0.97** | 0.63 | 0.85** | 0.69 | 0.87** | 0.73 | 0.99** |
| **SeA : Orth.A** | 1.00** | 0.92* | 1.00** | 0.02** | 1.00** | 0.41 | 1.00** | 0.94* | 1.00** | 0.94* | 1.00** |
| **SeA : N** | 1.00** | 0.99** | 1.00** | 0.01** | 1.00** | 0.68 | 0.99** | 0.76 | 1.00** | 0.94** | 1.00** |
| **RA : EA** | 0.62 | 0.57 | 0.46 | 0.34 | 0.70 | 0.64 | 0.62 | 0.58 | 0.67 | 0.63 | 0.67 |
| **RA : OA** | 0.96** | 0.72* | 0.97** | 0.15** | 0.94** | 0.59 | 0.90** | 0.75* | 0.97** | 0.91** | 0.99** |
| **RA : Orth.A** | 0.98** | 0.58 | 1.00** | 0.07** | 0.98** | 0.31 | 0.97** | 0.95** | 1.00** | 1.00** | 1.00** |
| **RA : N** | 1.00** | 0.87** | 1.00** | 0.03** | 0.99** | 0.61 | 0.97** | 0.82** | 1.00** | 0.99** | 1.00** |
| **EA : OA** | 0.88** | 0.65 | 0.85** | 0.30 | 0.90** | 0.46 | 0.73 | 0.59 | 0.90** | 0.82* | 0.95** |
| **EA : Orth.A** | 0.89* | 0.52 | 0.97** | 0.18 | 0.93** | 0.28 | 0.77 | 0.77 | 1.00** | 0.97* | 1.00** |
| **EA : N** | 0.99** | 0.85* | 0.99** | 0.16* | 0.99** | 0.49 | 0.78* | 0.69 | 1.00** | 0.98** | 1.00** |
| **OA : Orth.A** | 0.62 | 0.39 | 0.83* | 0.53 | 0.60 | 0.24 | 0.68 | 0.66 | 0.72 | 0.66 | 0.83* |
| **OA : N** | 0.80** | 0.71 | 0.91** | 0.41 | 0.79** | 0.50 | 0.67 | 0.57 | 0.82** | 0.78** | 0.92** |
| **Orth.A : N** | 0.72 | 0.74 | 0.64 | 0.32 | 0.72 | 0.78 | 0.47 | 0.42 | 0.74 | 0.72 | 0.67 |
| Values correspond to AUCs derived with binary ROC analysis. Significant AUCs (marked with asterisks) were defined  as (1) CI not crossing the midline, i.e. 0.5, and (2) p value (adjusted by false discovery rate) <0.05 (*) or <0.01 (**).  Significant AUCs <0.5 are underlined. Pairs are set up such that the presumably more inflamed sample group (diagnosis) is listed first, using the hierarchy SeA>RA>EA>OA>Orth.A>N. Abbreviations: EA, early arthritis; OA, osteoarthritis; Orth.A, orthopedic arthropathies; RA, rheumatoid arthritis; SeA, chronic septic arthritis. | | | | | | | | | | | |
